# Garcioligantone J and K, a pair of epimeric caged-polyprenylated xanthonoids from *Garcinia Oligantha*, inhibit the growth of lung cancer cells through ER stress-mediated apoptosis

**DOI:** 10.1016/j.pscia.2025.100065

**Published:** 2025-02-21

**Authors:** Lingyu Li, Hao Zheng, Qingying Liu, Dongmei Ren

**Affiliations:** a Key Laboratory of Chemical Biology (Ministry of Education), School of Pharmaceutical Sciences, Shandong University, 44 West Wenhua Road, Jinan, 250012, China; b School of Pharmaceutical Sciences, Shandong Xiandai University, China

**Keywords:** Garcioligantone J and K, Apoptosis, ER stress, ROS, Lung cancer

## Abstract

Garcioligantone J and K (GLJ and GLK) are a pair of isomers isolated from *Garcinia Oligantha* Merr. Herein, we described the structure elucidation including the absolute configurations of GLJ and GLK, explored and compared their anti-cancerous effects and underlying mechanism in A549 and NCI-H292 ​cells. The results indicated that GLJ and GLK are two isomers with different configuration at C-12, they inhibited cell proliferation and induced apoptosis in two lung cancer cell lines with almost the same extent. The induction of apoptosis by GLJ and GLK was demonstrated by DAPI and annexin-V-FITC/PI staining. Further investigation revealed increased Bax/Bcl-2 ratio, cleaved caspase-3, caspase-9 and PARP, loss of mitochondrial membrane potential (MMP) in cells, indicating that GLJ and GLK induced mitochondrial apoptosis. Increased GRP78, p-eIF2α and GADD153 manifested that endoplasmic reticulum (ER) stress was induced by GLJ and GLK. Meanwhile, upregulated reactive oxygen species (ROS) level was found and GLJ and GLK-induced ER stress and apoptosis could be attenuated by ROS scavenger NAC. Apoptosis induced by GLJ and GLK also could be alleviated by ER stress inhibitor 4-PBA. These showed that GLJ and GLK-induced apoptosis was mediated by ER stress relied on ROS generation. The efficacy of GLJ and GLK on lung cancer cell proliferation was further demonstrated in a zebrafish xenograft model. Collectively, the absolute configurations of GLJ and GLK were identified and they exerted lethal effects on lung cancer cells to the same extent via ROS-ER stress-mitochondrial apoptosis signaling, suggesting that GLJ and GLK might be used as potential modulating agents in lung cancer treatments.

## 1 Introduction

*Garcinia oligantha* is a species of tree that belongs to the genus *Garcinia*, family *Clusiaceae* (previously known as *Guttiferae*). Native to tropical regions, this plant is particularly found in Guangdong and Hainan province of China as well as north of Vietnam [1]. Previous phytochemical investigations have revealed that *G. oligantha* is rich in prenylated caged xanthones, which represents a unique class of structures with a tricyclo-4-oxa [4.3.1.0^3.7^] decan-2-one system [2,3]. Due to their interesting chemical structures and significant bioactivities, these caged xanthones have attracted increasing attentions in recent decades, highlighting their potential for further research and application.

During our research on the leaves and twigs of *G. oligantha*, a pair of epimeric caged-polyprenylated xanthonoids, garcioligantone J and K (GLJ and GLK) were isolated. These two compounds showed very high degree of similarities in their NMR data and CD spectrum, by using modified Mosher's method, they are finally identified as a pair of epimers with different configurations of C-12. GLJ and GLK displayed notable anti-proliferative effects against A549 and NCI-H292 lung cancer cells. Further investigations indicated that the compounds induced cell apoptosis, endoplasmic reticulum (ER) stress played key roles in the occurrence of apoptosis.

ER serves as a critical site within cells for synthesizing and modifying proteins and lipids [4]. When cells were exposed to a range of cytotoxic conditions, ER stress occurred. To counteract this stress, the cell initiates the unfolded protein response (UPR) signaling pathway. However, if the UPR becomes severe and prolonged, it can trigger cell apoptosis [5,6]. Recent findings suggest that during the process of apoptosis, the ER interacts with other organelles, such as mitochondria [7]. In this study, it was shown that both GLJ and GLK inhibited lung cancer cell growth to a similar degree. The cytotoxicity observed with GLJ and GLK was due to their induction of mitochondria-mediated cell apoptosis, and ER stress played a role in facilitating this apoptosis.

## 2 Materials and methods

### 2.1 Cells and culture

A549, NCI-H292 together with BEAS-2B cells were all sourced from Shanghai Cell Bank (Shanghai, China). NCI-H292 ​cells were grown in RPMI 1640 medium (Gibco, USA), while A549 and BEAS-2B cells were maitained in DMEM medium (Gibco, USA). All culture media were enriched with 10% (v/v) FBS (HyClone, USA).

### 2.2 Chemicals and reagents

Tunicamycin (TM) was purchased from Solarbio (Beijing, China). Cisplatin (CDDP), Z-VAD(OMe)-FMK (Z-VAD) and 4-phenylbutyric acid (4-PBA) were from Med Chem Express (NJ, USA). Annexin V-FITC/PI kit was acquired from BD Biosciences (San Jose, USA). CCCP (Carbonyl cyanide 3-chlorophenylhydrazone) was from Beyotime (Shanghai, China). DCFH-DA and DAPI were obtained from Sigma-Aldrich (St. Louis, MO, USA), and JC-1 was from Cayman Chemical.

Primary antibodies including Caspase-3 (19677-1-AP), Caspase-9 (10380-1-AP), Cleaved caspase-9 (10380-1-AP), and Cleaved caspase-3 (19677-1-AP), secondary antibodies including anti-rabbit IgG and anti-mouse IgG were all from Proteintech (USA). Primary antibodies including GADD153 (sc-7351), GRP78 (sc-166490), and β-tubulin (sc-5274) were from Santa Cruz (USA). Antibodies p-eIF2α (AP0692) and PARP (Cat# 9542) were from ABclonal (Wuhan, China) and Cell Signaling Technology (USA) respectively.

### 2.3 MTT assay

Cells were plated in 96-well plates at a concentration of 10,000 ​cells per well and left to incubate overnight. Following this, the cells were exposed to either DMSO, GLJ or GLK for specified durations. After the treatment period, 10 ​μl of a 5.0 ​mg/ml MTT solution was introduced into each well, and the plates were incubated for another 4 ​h. Subsequently, the medium was carefully aspirated, and DMSO was added to solubilize the resulting formazan crystals. The absorbance was then quantified at a wavelength of 570 ​nm.

### 2.4 Apoptosis assay

Cells were plated in 6-well plates and then treated with either DMSO, GLJ or GLK. After treatment, cells were stained the with Annexin V-FITC and PI. A FACS Calibur flow cytometer (BD Biosciences, USA) was used for analyzation [8].

### 2.5 DAPI staining

Cells grown in a 24-well plate were exposed to either DMSO, GLJ or GLK for 24 ​h. Following fixation, cells were treated with DAPI solution at a concentration of 2.5 ​μg/ml. The nuclei were observed by fluorescence microscope (Olympus IX71, Japan).

### 2.6 Immunoblot analysis

Sample buffer containing 50 ​mM Tris-HCl (pH 6.8), 2 ​% SDS, 10% glycerol, 100 ​mM DTT, and 0.1% bromophenol blue was used for the preparation of cell lysate. Equal protein quantities were then loaded and separated via SDS-PAGE gel electrophoresis. The separated proteins were subsequently transferred onto a nitrocellulose membrane. After blocking, the membrane was incubated with primary antibodies overnight at 4°C, followed by a 2 h exposure to HRP-conjugated secondary antibodies at room temperature. Finally, the signals were visualized using ECL reagents (Millipore, Billerica, MA, USA) [8]

### 2.7 Intracellular ROS measurement

Cells were exposed to DMSO, GLJ or GLK for 24 ​h, rinsed and treated with 5 ​μM DCFH-DA. Following this, cells were collected and suspended in PBS, then analyzed using a FACS Calibur flow cytometer with the excitation at 488 ​nm and the emission at 530 ​nm.

### 2.8 Mitochondrial membrane potential (MMP) measurement

MMP was assessed using JC-1 staining. Cells grown in 6-well plates were exposed to DMSO, GLJ or GLK for 24 ​h. Following this, cells were treated with 2 ​μM JC-1 for 30 ​min. Fluorescence microscopy was employed to capture the resulting images.

### 2.9 Zebrafish xenograft model

Wild-type AB zebrafish were sourced from the China Zebrafish Resource Center. Establishment of a zebrafish xenograft model was following established protocols [9,10]. Briefly, mature zebrafish were naturally mated to obtain fertilized eggs, which were then incubated at 28°C in an embryonic medium consisting of 5.0 ​mM NaCl, 0.17 ​mM KCl, 0.4 ​mM CaCl_2_, and 0.16 ​mM MgSO_4_. Embryos that were 48 ​h post-fertilization (hpf) were utilized for the experiments. A549 cells were labeled with 2 ​μM of the cell tracker CM-Dil prior to harvesting. Each zebrafish embryo, anesthetized with tricaine, received a microinjection of 200 labeled cells into the yolk sac. Four hours post-injection, the surviving zebrafish that bore tumors were sorted into various groups, with 20 zebrafish allocated to each group. Following a 48 h treatment with GLJ/K (0.5 and 1.0 ​μM) or CDDP via soaking, the zebrafish were examined and imaged using a confocal microscope (Olympus FV1000, Japan). Tumor growth inhibition was assessed by measuring fluorescence intensity, which was quantified using Image J software.

### 2.10 Statistical analysis

The data are expressed as mean ​± ​standard deviation (SD). Statistical analysis was conducted using a one-way ANOVA, followed by a Bonferroni post hoc test for multiple comparisons, to assess significant differences between the groups. *p* ​< ​0.05 was deemed statistically significant.

## 3 Results

### 3.1 Structure elucidation of GLJ and GLK

**Fig. 1:**
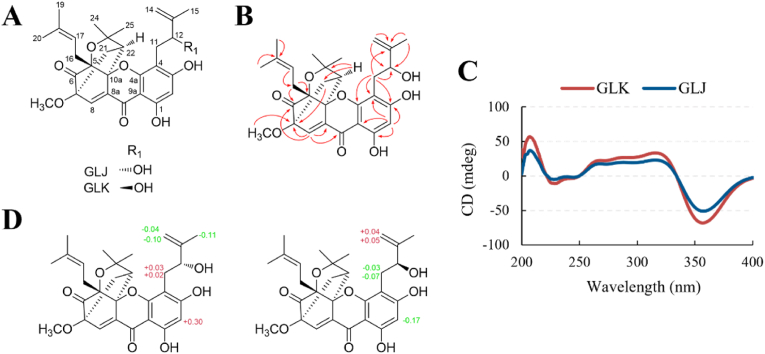
Structure elucidation of GLJ and GLK. (A) Chemical structures of GLJ and GLK. (B) Key HMBC correlations of GLJ and GLK. (C) ECD curves of GLJ and GLK. (D) Modified Mosher's method was used for the determination of C-12 configuration of GLJ and GLK.

GLJ and GLK (Fig. 1A) were obtained from the ethanol extract of *G. oligantha* through the use of chromatography methods including silica-gel column chromatography, sephadex LH-20 column chromatography and HPLC. The purities of GLJ and GLK were 100% and 94% analyzed by HPLC. The ^1^H and ^13^C spectra of the two compounds were almost identical except for some minor differences (Table 1). Typical signals at *δ*_H_ 2.55 (1H, d, *J* ​= ​10.0 ​Hz, H-22), 2.33 (1H, d, *J* ​= ​13.0 ​Hz, H_a_-21), 1.64 (1H, dd, *J* ​= ​13.0, 10.0 ​Hz, H_b_-21) and 7.50 (1H, s, H-8) indicated the existence of a tricyclo-4-oxa[4.3.1.0^3.7^]decan-2-one system, which is the representative structural moiety of the caged-polyprenylated xanthonoids [2,3]. With the aid of HMBC, HMQC and ^1^H–^1^H COSY data (Fig. 1B), the structures of GLJ and GLK were established except for the absolute configurations of C-12. The ECD curves of GLJ and GLK are also very similar, suggesting the configurations of C-12 exerted little effect on the ECD effects (Fig. 1C). Ultimately, modified Mosher's method was used for the determination of C-12 configuration, GLJ was 12*R* and GLK was 12*S* (Fig. 1D). All of the abovementioned spectroscopic data are consistent with the data reported, the structure of GLJ and GLK were elucidated undoubtedly [11].

**Table 1:** ^1^H (600 ​MHz) and^13^C (150 ​MHz) NMR data of GLJ and GLK (*δ* in ppm, *J* in Hz).

No.	GLJ		GLK	
	*δ*_C_	*δ*_H_	*δ*_C_	*δ*_H_
1	164.1		164.1	
2	98.9	6.17, s	98.9	6.15, s
3	166.7		166.9	
4	105.2		105.1	
4a	158.6		158.5	
5	84.4		84.5	
6	202.0		201.9	
7	85.1		84.9	
8	134.7	7.50, s	134.4	7.48, s
8a	132.1		131.9	
9	179.1		178.9	
9a	101.3		101.1	
10a	89.6		89.5	
11	29.8	3.22, d (15.0)	29.1	3.15, d (15.0)
		2.71, dd (15.0, 10.0)		2.80, dd (15.0, 10.0)
12	78.3	4.40, d (10.0)	78.7	4.35, d (10.0)
13	146.6		145.9	
14	112.3	5.11, s	113.7	4.91, s
		4.94, s		4.90, s
15	18.0	1.86, s	17.0	1.88, s
16	30.8	2.45, dd (15.0, 10.0)	28.9	2.48, dd (15.0, 10.0)
17	117.5	4.42, m	117.3	4.40, m
18	135.7		135.7	
				
19	25.7	1.39, s	25.7	1.36, s
20	17.1	1.10, s	16.9	1.00, s
21	31.1	2.33, d (13.0)	31.2	2.34, d (13.0)
		1.64, dd (13.0, 10.0)		1.66, dd (13.0, 10.0)
22	50.1	2.55, d (10.0)	49.9	2.55, d (10.0)
23	83.5		83.6	
24	29.3	1.62, s	30.3	1.56, s
25	29.1	1.28, s	29.0	1.27, s
7-OCH_3_	54.1	3.63, s	54.1	3.62, s
1-OH		12.54, s		12.57, s

Measured in CDCl_3_.

### 3.2 GLJ and GLK inhibited proliferation of lung cancer cells

**Fig. 2:**
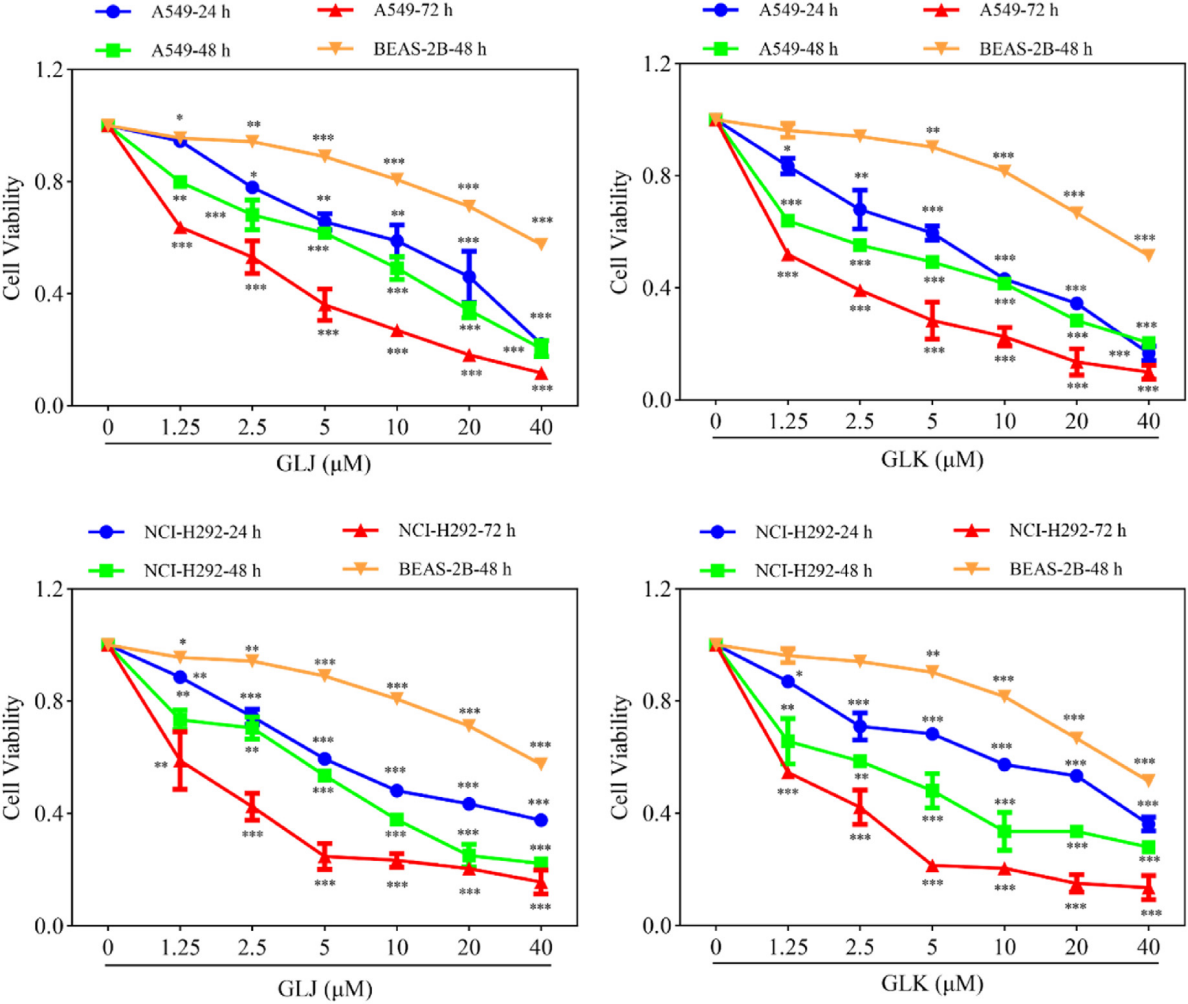
GLJ and GLK inhibited proliferation of A549 and NCI-H292 ​cells. A549, NCI-H292, and BEAS-2B cells underwent GLJ/GLK treatment at indicated concentrations and durations, followed by MTT assay for cell viability assessment. Data were expressed as mean ​± ​SD, n ​= ​3. ∗*p* ​< ​0.05, ∗∗*p* ​< ​0.01, and ∗∗∗*p* ​< ​0.001.

The anti-proliferative properties of GLJ and GLK were initially evaluated first. Two lung cancer cell lines, A549 and NCI-H292, were exposed to varying concentrations of GLJ and GLK (ranging from 0 to 40 ​μM, specifically 0, 1.25, 2.5, 5, 10, 20, and 40 ​μM) over periods of 24, 48, and 72 ​h. Cell viability was then measured using the MTT assay. As depicted in Fig. 2, both GLJ and GLK reduced cell viability in a dose-dependent manner. At the 24 ​h and 48 ​h time points, GLJ and GLK exhibited similar comparable cytotoxic effects. However, after 72 ​h of treatment, more significant effects were observed. Based on the results from 48 ​h treatment, the IC_50_ values of GLJ were 8.84 ​μM and 6.50 ​μM for A549 and NCI-H292 ​cells, respectively. In contrast, the IC_50_ values of GLK were 5.48 ​μM and 5.81 ​μM for A549 and NCI-H292 ​cells, respectively. According to these MTT assay data, it appears that GLJ and GLK have nearly identical inhibitory efficacy on the proliferation of lung cancer cells, indicating that the configuration of C-12 did not significantly impact their cytotoxicity.

### 3.3 GLJ and GLK induced apoptosis in A549 and NCI-H292 ​cells

**Fig. 3:**
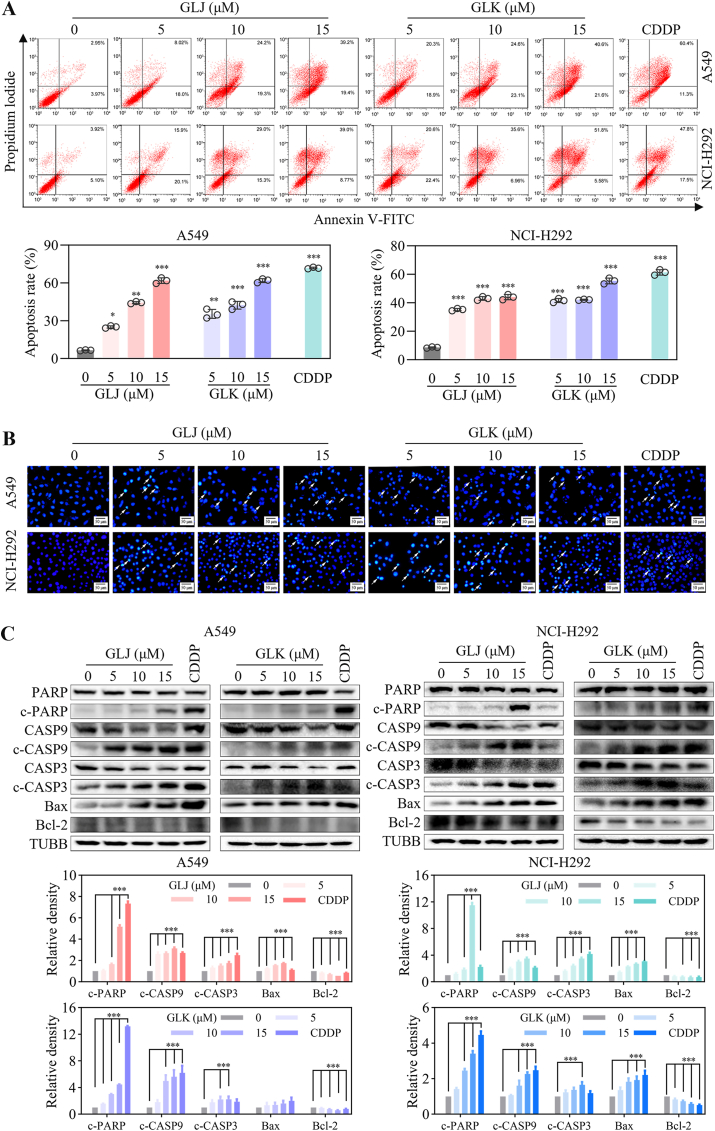
GLJ and GLK induced apoptosis in A549 and NCI-H292 ​cells. (A) Cells were exposed to GLJ and GLK (5, 10, and 15 ​μM) for 48 ​h, subsequently stained using Annexin V/PI, and analyzed via flow cytometry. (B) Following a 24 ​h treatment with GLJ and GLK (5, 10, and 15 ​μM) or CDDP (20 ​μM), nuclear morphology was examined under fluorescence microscopy after DAPI staining, with a scale bar of 20 ​μm provided for reference. (C) After incubation with GLJ and GLK (0, 5, 10, and 15 ​μM) or CDDP (20 ​μM) for 24 ​h, cell lysates were obtained and analyzed through immunoblotting for cleaved-PARP (c-PARP), cleaved-caspase-9 (c-CASP9), cleaved-caspase-3 (c-CASP3), Bax, and Bcl-2, using β-tubulin (TUBB) as a loading control. The data are expressed as mean ​± ​SD from three separate experiments. ∗*p* ​< ​0.05, ∗∗*p* ​< ​0.01 and ∗∗∗*p* ​< ​0.001 compared with the control group.

Apoptosis, often referred to as the body's built-in mechanism for programmed cell death, plays a pivotal role in maintaining cellular balance [12]. To examine whether GLJ and GLK were triggering cell death via apoptosis, we stained A549 and NCI-H292 ​cells treated with GLJ and GLK using annexin V-FITC/PI and DAPI. Flow cytometry showed a marked increase in annexin V-positive cells in both cell lines after GLJ and GLK treatment, which points toward apoptosis happening (Fig. 3A). As shown in Fig. 3B, the nuclei of A549 and NCI-H292 ​cells treated with GLJ and GLK were more condensed and fragmented compared to the control group. These findings indicated that GLJ and GLK induced lung cancer cell death predominantly through the apoptotic pathway.

**Fig. 4:**
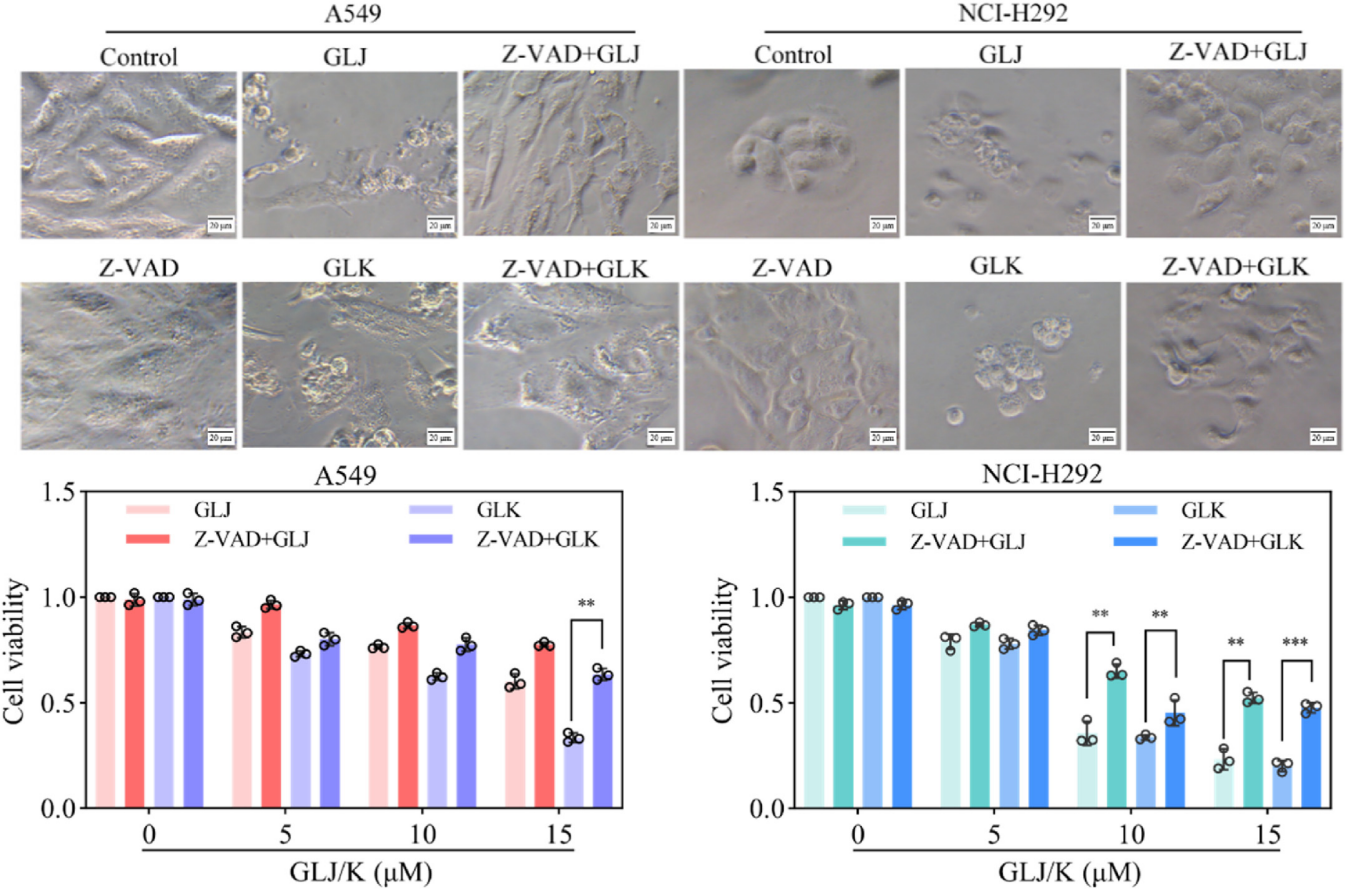
GLJ and GLK-induced cytotoxicity was significantly reversed by pan-caspase inhibitor Z-VAD in A549 and NCI-H292 ​cells. The A549 and NCI-H292 ​cells were subjected to a 2 ​h pretreatment with or without Z-VAD (20 ​μM) before introducing GLJ/K (15 ​μM) or DMSO. Phase-contrast images were obtained after 24 ​h (upper). A scale bar of 20 ​μm is provided for reference. Cell viability was subsequently assessed using the MTT assay after 48 ​h (lower). Values were shown as mean ​± ​SD (n ​= ​3). ∗∗*p* ​< ​0.01 and ∗∗∗*p* ​< ​0.001.

The sequential activation of caspase family proteins is regarded as an essential condition for the process of apoptosis [13]. To explore the role of caspase activation in the apoptosis induced by GLJ and GLK, we measured the protein levels of caspase-9, caspase-3, and PARP. The findings revealed that treatment with GLJ and GLK resulted in elevated levels of cleaved caspase-9, caspase-3, and PARP. Furthermore, treatment with GLJ and GLK was found to upregulate the expression of the pro-apoptotic protein Bax while downregulating the expression of the anti-apoptotic protein Bcl-2 in both A549 and NCI-H292 ​cell lines (Fig. 3C). GLJ and GLK-induced cytotoxicity was significantly reversed by 2 ​h pretreatment with pan-caspase inhibitor Z-VAD prior to GLJ and GLK treatment (Fig. 4). These results provide additional evidence suggesting that GLJ and GLK trigger apoptosis likely via the intrinsic mitochondrial pathway.

### 3.4 GLJ and GLK induced loss of MMP and accumulation of ROS in A549 and NCI-H292 ​cells

The destabilization of the mitochondrial membrane is a pivotal factor in triggering the intrinsic pathway of mitochondrial apoptosis [14]. To assess changes in MMP within A549 and NCI-H292 ​cells, JC-1 staining was used. This dye specifically accumulates in mitochondria, and when MMP is compromised, it transitions into monomers that produce green fluorescence. In contrast, under stable MMP conditions, JC-1 assembles into aggregates that emit red fluorescence. By analyzing the ratio of red to green fluorescence, scientists can effectively gauge alterations in MMP.

**Fig. 5:**
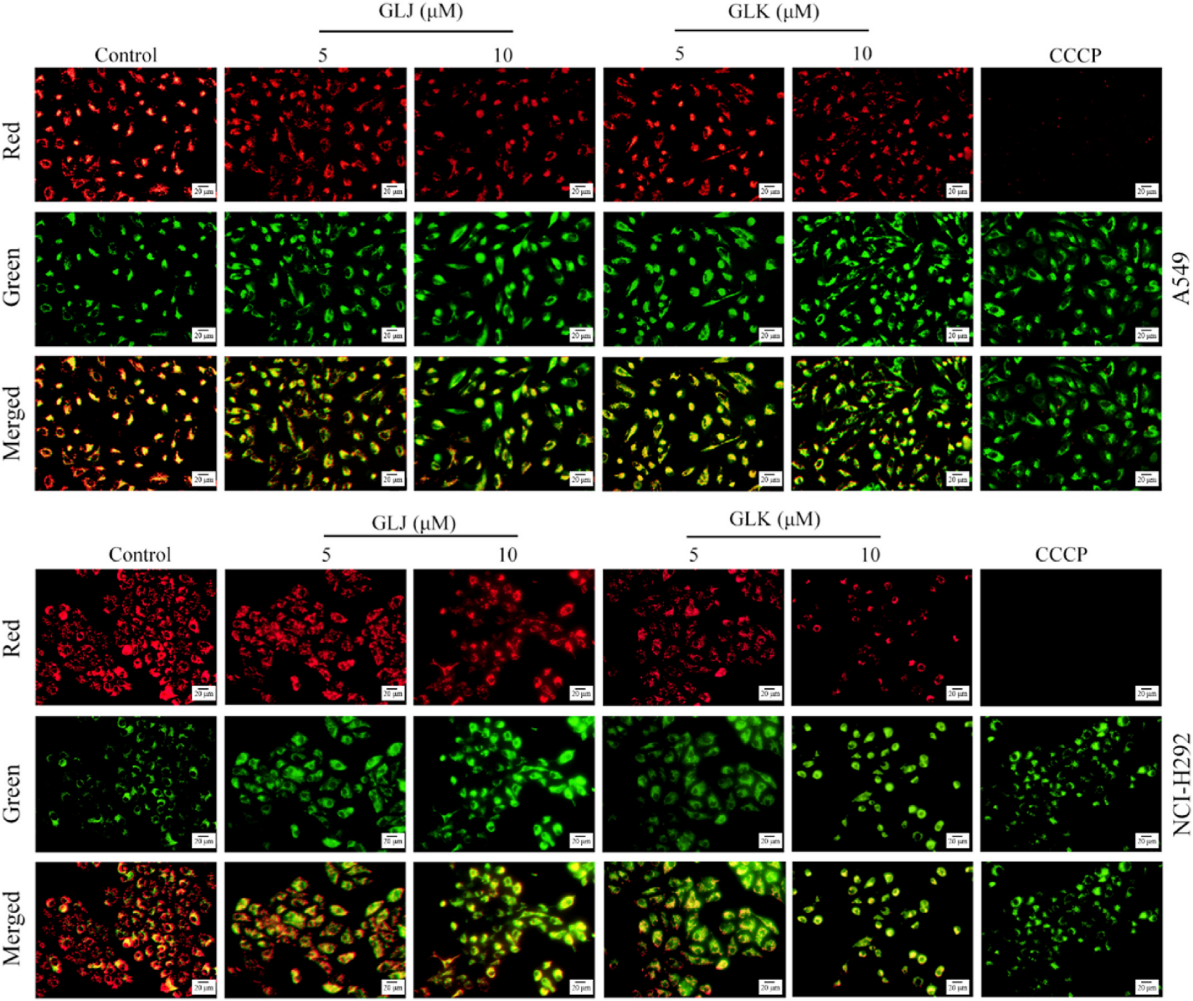
GLJ and GLK induced loss of MMP in A549 and NCI-H292 ​cells. Cells were treated with DMSO (control), GLJ and GLK (5 and 10 ​μM) or CCCP (15 ​μM) for 24 ​h, and MMP changes were then tracked using JC-1 staining and visualized through fluorescence microscopy. CCCP acted as the positive control in this experiment. The scale bar is indicated as 20 ​μm.

As illustrated in Fig. 5, cells exposed to DMSO showed a marked increase in the red/green fluorescence intensity ratio for both A549 and NCI-H292 ​cells. In contrast, treatment with GLJ and GLK resulted in a lower red/green fluorescence intensity ratio in both cell lines. CCCP, used as a positive control, practically wiped out the red fluorescence, causing a dramatic drop. These findings suggest that treatment with GLJ and GLK led to mitochondrial depolarization and a loss of MMP in both A549 and NCI-H292 ​cells.

**Fig. 6:**
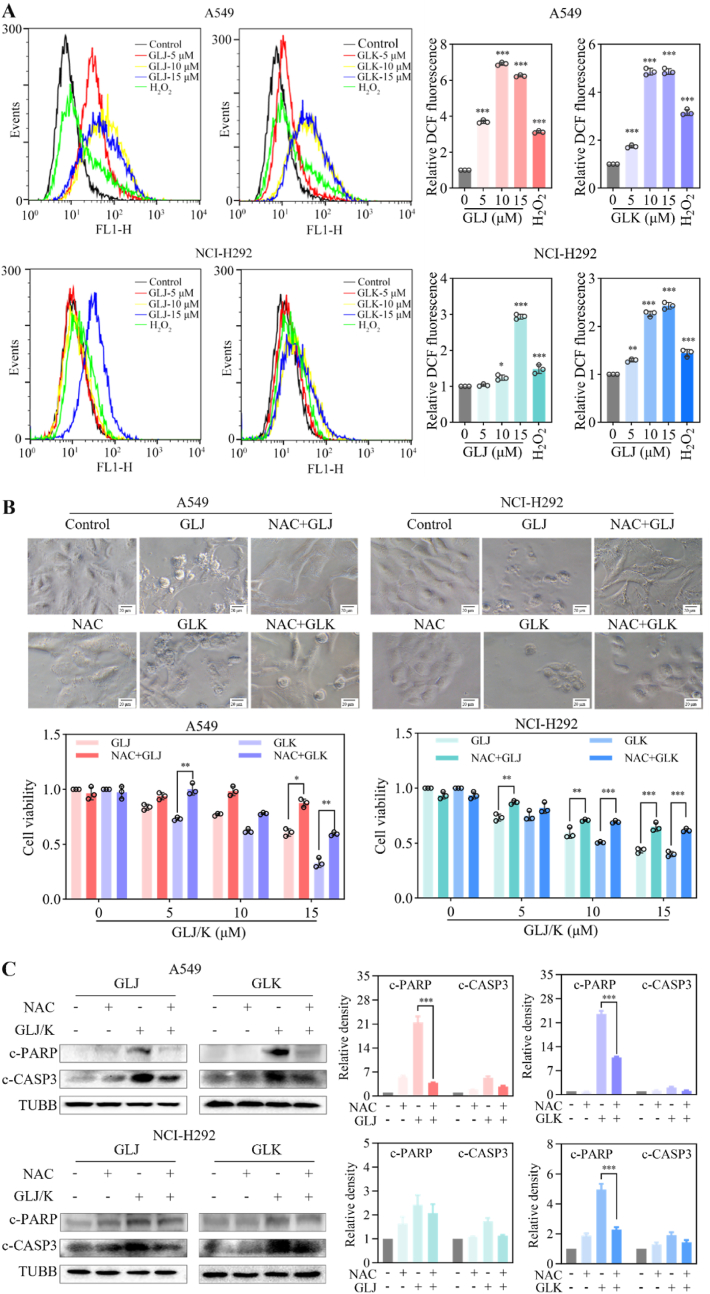
GLJ and GLK induced accumulation of ROS in A549 and NCI-H292 ​cells. (A) Cells were exposed to GLJ and GLK at concentrations of 0, 5, 10, and 15 ​μM for a duration of 24 ​h, and the levels of ROS were assessed using flow cytometry, with H_2_O_2_ serving as the positive control. (B) The A549 and NCI-H292 ​cells underwent a 2 ​h pretreatment with or without NAC (2 ​mM) before the introduction of GLJ and GLK (15 ​μM) or DMSO. Phase-contrast images were captured after 24 ​h (upper), with a scale bar representing 20 ​μm. The MTT assay was employed to evaluate cell viability after 48 ​h (lower). (C) Following the 24 ​h treatment, cell lysates were harvested and analyzed using immunoblotting to detect c-PARP and c-CASP3 levels. Data were presented as the mean ​± ​SD (n ​= ​3). ∗*p* ​< ​0.05, ∗∗*p* ​< ​0.01, and ∗∗∗*p* ​< ​0.001.

Mitochondrial dysfunction, along with the ensuing release of reactive oxygen species (ROS), is pivotal in triggering apoptosis [15]. Using DCFH-DA staining followed by flow cytometry analysis, we detected a marked increase in ROS levels within A549 and NCI-H292 ​cell lines following treatment with GLJ and GLK, as demonstrated by the heightened fluorescence intensity (Fig. 6A). When co-treated with N-acetyl-L-cysteine (NAC), a known ROS scavenger, the upregulation of cleaved PARP and cleaved caspase-3 induced by GLJ/K was effectively inhibited (Fig. 6C). Additionally, NAC counteracted the alterations in cell morphology and the cell death prompted by GLJ and GLK (Fig. 6B). These findings imply that the generation of ROS plays a crucial role in mediating the cytotoxic effects associated with GLJ and GLK.

### 3.5 GLJ and GLK induced ER stress in A549 and NCI-H292 ​cells

**Fig. 7:**
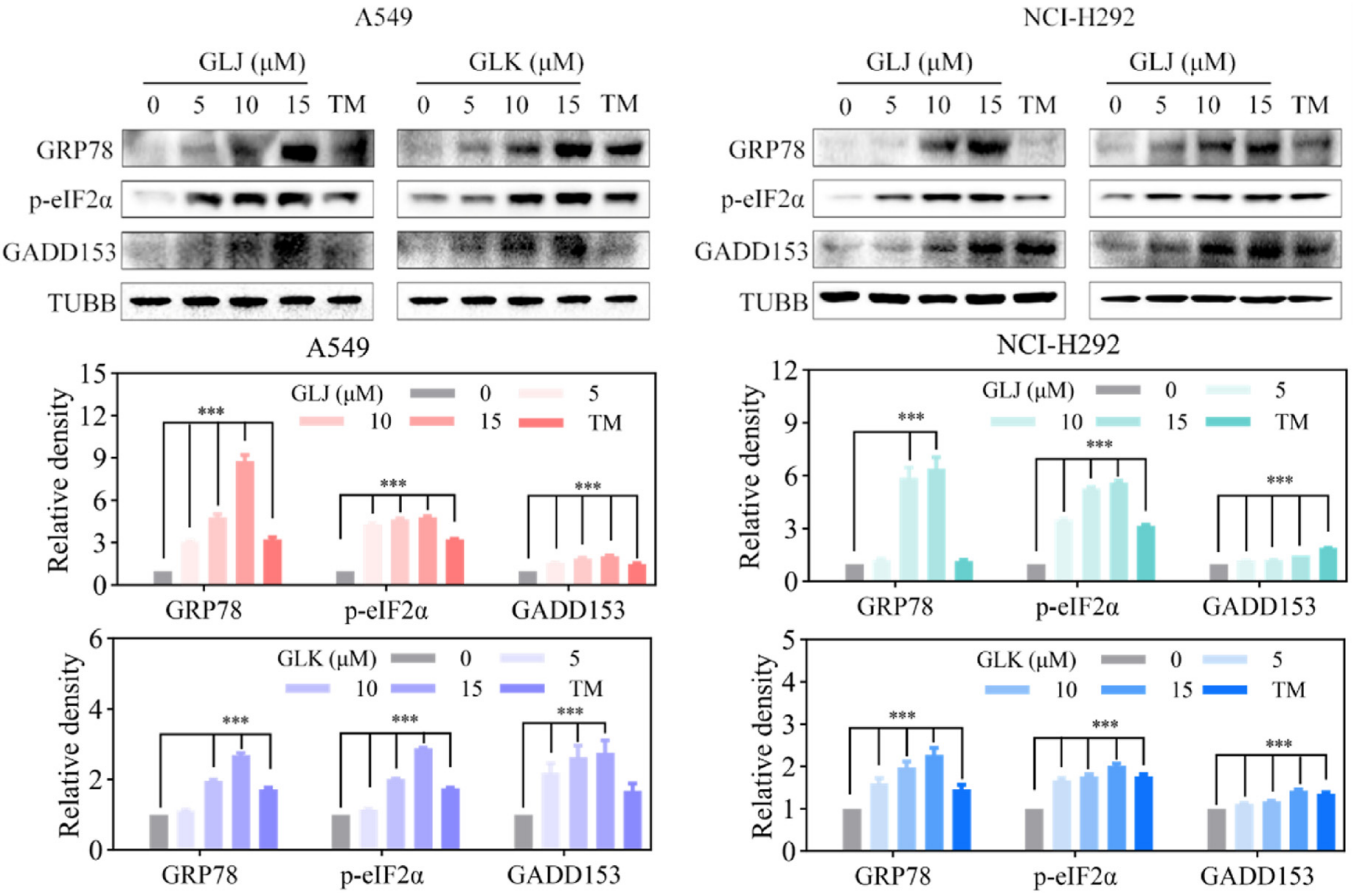
GLJ and GLK induced ER stress in A549 and NCI-H292 ​cells. Cells were exposed to GLJ and GLK (0, 5, 10, and 15 ​μM) or TM (3 ​μM) for 16 ​h, followed by immunoblotting of lysates for ER stress and UPR-related proteins. Data were presented as the mean ​± ​SD (n ​= ​3). ∗∗∗*p* ​< ​0.001.

ER stress arises from various cytotoxic stimuli and triggers the UPR pathway. When ER stress is severe and persistent, beyond the cell's ability to adapt, the UPR can initiate apoptosis [6]. In experiments involving A549 and NCI-H292 ​cell lines treated with GLJ and GLK, there was a notable increase in the expression of the ER chaperone glucose-regulated protein 78 (GRP78), which rose significantly in a dose-dependent fashion. (Fig. 7). Additionally, the activation of downstream UPR signaling proteins was observed. Following treatment with GLJ and GLK, both A549 and NCI-H292 ​cell lines exhibited a marked increase in the protein levels of phosphorylated eukaryotic initiation factor 2α (p-eIF2α) and C/EBP-homologous protein (CHOP/GADD153). Tunicamycin (TM) served as the positive control in these experiments. These findings indicate that GLJ and GLK triggered ER stress and subsequently activated the UPR signaling pathway in the both cell lines.

### 3.6 GLJ and GLK-induced apoptosis was mediated by ER stress

**Fig. 8:**
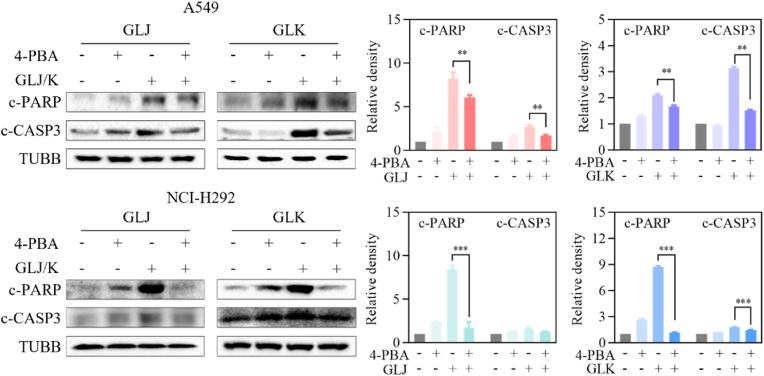
GLJ and GLK-induced apoptosis was mediated by ER stress. A549 and NCI-H292 ​cells underwent a 2 ​h pretreatment with or without 4-PBA (5 ​mM) before introducing GLJ and GLK (15 ​μM) or DMSO. Following a 24 ​h incubation, cell lysates were harvested and analyzed through immunoblotting for c-PARP and c-CASP3. Data were shown as the mean ​± ​SD (n ​= ​3). ∗∗*p* ​< ​0.01, ∗∗∗*p* ​< ​0.001.

To evaluate the influence of ER stress on GLJ/GLK-induced programmed cell death, cells received a 4-PBA pretreatment to suppress ER stress. Following this pretreatment, two key markers of apoptosis, cleaved PARP and cleaved caspase-3, were analyzed using immunoblotting. The results depicted in Fig. 8 indicate that 4-PBA pretreatment successfully blocked the phosphorylation of PARP and caspase-3 that was otherwise induced by GLJ and GLK treatments. This result indicates that ER stress is crucial in mediating the apoptotic effects of GLJ and GLK in A549 and NCI-H292 ​cells.

### 3.7 GLJ and GLK inhibited the growth of lung cancer cells in zebrafish xenograft model

**Fig. 9:**
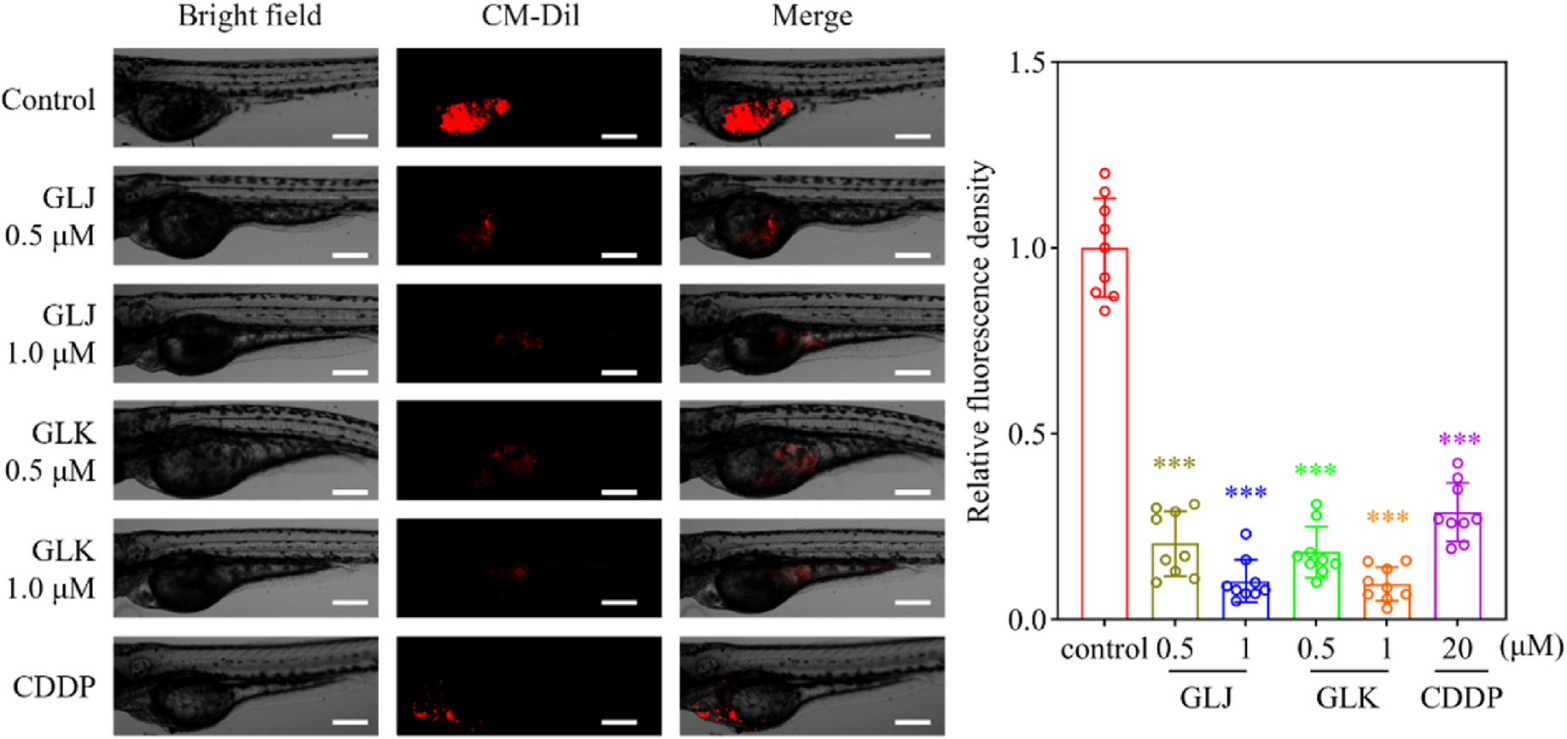
GLJ and GLK inhibited the growth of lung cancer cells in zebrafish xenograft model. Images depicting tumor-infected zebrafish subjected to DMSO, GLJ, GLK, or CDDP for 48 ​h illustrate the anti-proliferative effects, evidenced by the area and intensity of red fluorescence (left). A scale bar of 500 ​μm is provided. The fluorescence intensity measurements were processed using Image J software (right). Results are presented as mean ​± ​SD (n ​= ​9). Compared with control, ∗∗∗*p* ​< ​0.001.

The zebrafish xenograft model has emerged as a valuable tool to gauge how well potential cancer drugs work at stopping tumor cells from multiplying, particularly because it significantly reduces the amount of drug needed for testing. Given the limited availability of GLJ and GLK, we employed an A549-derived zebrafish model to evaluate the *in vivo* impact of these two compounds on lung cancer cell proliferation. After a 48 h treatment with GLJ and GLK at concentrations of 0.5 ​μM and 1.0 ​μM, respectively, a notable decrease in the tumor formation capacity of A549 ​cells was observed in the zebrafish xenograft model (Fig. 9).

## 4 Discussion

In 1972, Kerr first described apoptosis as a unique type of programmed cell death, characterized by specific morphological alterations, including the loss of specialized cellular structures such as microvilli and protrusions, chromatin condensation, nuclear pyknosis, and the formation of apoptotic bodies [16]. At the biochemical level, apoptosis is characterized by a cascade of critical events, such as the disruption of mitochondrial function, which results in a drop in ATP synthesis. This is accompanied by the opening of mitochondrial permeability transition pores and the activation of endonucleases that fragment DNA. Additionally, there's a noticeable increase in the expression of proteins linked to apoptosis, particularly those belonging to the caspase, PARP, and Bcl-2 families. These processes collectively drive the programmed cell death pathway [17,18]. In addition, caspase inhibitors block these processes, and phosphatidylserine externalization on the plasma membrane serves as a hallmark of apoptosis [19].

Apoptosis is typically categorized into intrinsic and extrinsic pathways [20]. With the further application of conventional chemotherapy drugs, tumor resistance become an increasing concern. The ER play a central role in protein folding, and prolonged ER stress activate apoptosis through the UPR. Herein, targeting ER-dependent apoptosis present a potential strategy to overcome tumor resistance for chemotherapy [21,22]. Isoprenylated xanthone derivatives GLJ and GLK were identified as potent inhibitors of tumor cell proliferation in preliminary screenings. In this study, we compared their antiproliferative effects in lung cancer cell lines (A549 and NCI-H292) and investigated the underlying mechanisms.

Both GLJ and GLK exhibited similar antiproliferative activity in A549 and NCI-H292 ​cells (Fig. 2). Mechanistic investigations revealed that treatment with GLJ/K induced apoptosis, a conclusion supported by Annexin V/PI and DAPI staining assays, which highlighted the characteristic morphological and biochemical markers of programmed cell death (Fig. 3). Further analysis indicated that GLJ/K activated the intrinsic mitochondrial apoptosis pathway, evidenced by the upregulation of cleaved PARP and caspase-3, a heightened Bax/Bcl-2 ratio, and a reduction in MMP (Fig. 3, Fig. 5). These findings collectively underscore the involvement of mitochondrial dysfunction in the apoptotic cascade triggered by GLJ/K.

Moreover, pretreatment with NAC, a well-known ROS scavenger, effectively mitigated the GLJ/K-induced upregulation of cleaved PARP and caspase-3, while also reversing the associated changes in cell morphology and cytotoxicity. This observation strongly implicates ROS as critical mediators of the apoptotic process induced by GLJ/K (Fig. 6). In addition to mitochondrial apoptosis, GLJ/K was found to induce ER stress and the UPR, as indicated by the elevated expression of key markers such as GRP78, p-eIF2α, and GADD153 (Fig. 7). The role of ER stress in GLJ/K-induced apoptosis was further corroborated by the use of 4-PBA, an ER stress inhibitor, which attenuated the activation of cleaved PARP and caspase-3, thereby confirming the involvement of this pathway in the apoptotic mechanism (Fig. 8).

Intriguingly, the administration of GLJ/K demonstrated a marked reduction in the tumorigenic potential of A549 ​cells, as evidenced by the zebrafish xenograft model (Fig. 9). Specifically, the treated group exhibited a substantial decrease in tumor volume and metastatic spread compared to the control group, highlighting the compound's efficacy in inhibiting cancer progression. The results indicate that GLJ/K holds potential as a promising candidate for anticancer treatment. However, extensive and comprehensive research is essential to fully elucidate its therapeutic potential and effectiveness in overcoming chemotherapy resistance. Future studies might focus on exploring its impact on tumor microenvironment dynamics and immune response modulation, which will provide a more complete understanding of its therapeutic value.

## 5 Conclusions

In summary, GLJ/K, which is derived from G. *Oligantha*, has been recognized as a trigger for apoptosis. ROS-dependent ER stress and consequent UPR contributed to GLJ/K-induced apoptosis, this might be related to the mechanism of action of GLJ/K. Our findings indicate that GLJ/K has the potential to emerge as a promising candidate for anticancer treatments in the future.

## CRediT authorship contribution statement

**Lingyu Li:** Writing – original draft, Validation, Software, Project administration, Methodology, Investigation, Formal analysis, Data curation, Conceptualization. **Hao Zheng:** Software, Formal analysis, Data curation. **Qingying Liu:** Software, Methodology, Investigation. **Dongmei Ren:** Writing – review & editing, Validation, Supervision, Resources, Funding acquisition.

## Data availability

Data will be made available on request.

## Ethics approval

The zebrafish were handled in accordance with the Guide for the Care and Use of Laboratory Animals. All experimental protocols were meticulously reviewed and approved by the Ethics Committee for Pharmaceutical Sciences at Shandong University (approval number: IACUC-2024220-14).

## Funding information

This work was financially supported by the 10.13039/501100001809National Natural Science Foundation of China (No. 22277067) and the 10.13039/100014103Key Research and Development Program of Shandong Province (No. 2018GSF118085).

## Declaration of competing interest

The authors declare that they have no known competing financial interests or personal relationships that could have appeared to influence the work reported in this paper.
